# SARS-CoV-2 B.1.1.529 (Omicron) Variant Causes an Unprecedented Surge in Children Hospitalizations and Distinct Clinical Presentation Compared to the SARS-CoV-2 B.1.617.2 (Delta) Variant

**DOI:** 10.3389/fped.2022.932170

**Published:** 2022-06-27

**Authors:** Jessica Taytard, Blandine Prevost, Aurélie Schnuriger, Guillaume Aubertin, Laura Berdah, Lauren Bitton, Audrey Dupond-Athenor, Guillaume Thouvenin, Nadia Nathan, Harriet Corvol

**Affiliations:** ^1^Pediatric Pulmonology Department, APHP Hôpital Trousseau, Sorbonne Université, Paris, France; ^2^Sorbonne Université, Inserm UMR_S 1158, Paris, France; ^3^Sorbonne Université, Centre de Recherche Saint Antoine (CRSA), Inserm UMR_S938, Paris, France; ^4^Virology Department, APHP Hôpital Trousseau, Sorbonne Université, Paris, France; ^5^Sorbonne Université, Inserm UMR_S933, Paris, France

**Keywords:** COVID-19, children, SARS-CoV-2, delta variant, omicron variant, hospitalization

## Abstract

**Background:**

In the midst of successive waves of SARS-CoV-2 variants, the B.1.1.529 (omicron) variant has recently caused a surge in pediatric infections and hospitalizations. This study aimed to describe and compare the symptoms, explorations, treatment and evolution of COVID-19 in hospitalized children during the successive B.1.617.2 (delta) and B.1.1.529 (omicron) waves.

**Methods:**

This observational study was performed in the Pediatric Pulmonology Department of a University Hospital in Paris, France. All hospitalized children aged between 0 and 18 years who tested positive for SARS-CoV-2 using reverse transcription polymerase chain reaction (RT-PCR) in nasopharyngeal swabs from July 15th to December 15th 2021 (delta wave), and from December 15th 2021 to February 28th 2022 (omicron wave) were included.

**Results:**

In total, 53 children were included, 14 (26.4%) during the delta wave and 39 (73.6%) during the omicron wave (almost three times as many hospitalizations in half the time during the latter wave). During the omicron wave, hospitalized patients were mostly aged < 5 years (90 vs. 71% of all the children during omicron and delta waves, respectively), and tended to have fewer underlying conditions (56 vs. 79% during omicron and delta waves, respectively, *p* = 0.20). The omicron variant was also responsible for a different clinical presentation when compared to the delta variant, with significantly higher and often poorly tolerated temperatures (*p* = 0.03) and increased digestive symptoms (*p* = 0.01). None of the three patients who were older than 12 years were fully vaccinated.

**Conclusion:**

The dramatic increase in the hospitalization of children with COVID-19 and the modification of the clinical presentation between the latest delta and omicron waves require pediatricians to remain vigilant. It should also encourage caregivers to ensure vaccination in children older than 5 years, for whom the BNT162b2 COVID-19 vaccine has been deemed safe, immunogenic, and effective.

## Introduction

In France and throughout the world, the surge in coronavirus diseases (COVID-19) caused by the variant of concern B.1.1.529 (omicron), reached a peak that was five to six times higher than that caused by any of the previous severe acute respiratory syndrome coronavirus 2 (SARS-CoV-2) variants (1–3). After an important decrease in the SARS-CoV-2 circulation in June 2021, the French public health agency observed a progression in infections due to the B.1.617.2 (delta) variant that began in mid-July (4). The later switch between the delta and omicron variants began in mid-December 2021 (5). Whereas the incidence of COVID-19 had been far lower in children than in adults, it multiplied in children more than eight times during the period of this change in the SARS-CoV-2 virus variant. Specifically, the incidence of COVID-19 increased from 634/100,000 in the age groups 0–9 and 10–19 years at the delta-wave peak (week 49, 2021) to 4,877 and 6,828/100,000, respectively, in the same age groups at the omicron-wave peak (week 3, 2022) (4).

However, concerns about the high infectivity of the omicron variant have been balanced by its apparent lower severity in adults, with less severe symptoms and decreased hospitalization rates (2, 6, 7). This reduction in disease severity has partly been attributed to the widespread use of COVID-19 vaccines in adults (8, 9).

In contrast, pediatricians observed a surge in pediatric hospitalizations due to COVID-19 during the omicron wave (5, 10, 11). In the United States and South Africa, the peak of child hospitalizations resulted in a patient load that was four times higher than during the delta wave, with the largest increase occurring in children under 4 years of age (12, 13). Further, more children needed hospitalization in the intensive care unit (ICU) and/or invasive ventilation (12, 13). Interestingly, the monthly hospitalization rate in children aged 12–17 years was six times higher in non-vaccinated patients than in fully vaccinated patients (13). Compared to the delta variant, the omicron virus appears to have a predilection for the upper respiratory airways and digestive tract (5, 12). Reports have also described atypical cases of convulsions and cerebral venous thrombosis in children, making this a variant of concern, especially for pediatricians (14, 15).

To date, few studies have compared children with SARS-CoV-2 infection during the delta and omicron waves. Moreover, for the development of vaccines for children, it is important to precisely describe how children are affected by successive waves (16). Therefore, this study aimed to describe and compare the symptoms, explorations, treatment, and evolution of COVID-19 in children during the delta and omicron waves.

## Patients and Methods

This observational study was performed in the Pediatric Pulmonology Department of the University Hospital Trousseau, Assistance Publique Hôpitaux de Paris (APHP) Paris, France. According to the information on SARS-CoV-2 circulation in France, the arrival of the B.1.617.2 (delta) variant began in mid-July 2021 and that of the B.1.1.529 (omicron) variant began in mid-December 2021 (5). As such, patients between 0 and 18 years of age hospitalized in this department for COVID-19 between July 15th 2021 (arrival of the delta wave) and February 28th 2022 (end of the omicron wave) were identified using the hospital's “*Programme de Médicalisation des Systèmes d'Information”* (PMSI) database. This allowed for an exhaustive search of all children testing positive for SARS-CoV-2 by real-time reverse transcription polymerase chain reaction (RT-PCR) using nasopharyngeal swabs, who were admitted to this hospital. The study was approved by the local ethics committee of our institution, which waived the need for patients' consent (Study PED_COVID N°20200717191204).

Patient information was retrieved from medical records, including COVID-19 transmission history, clinical, biological (blood tests and viral RT-PCR findings) and radiological information, and the medical evolution. Considering the SARS-CoV-2 variant circulation in France, children hospitalized between July 15th 2021 and December 15th 2021 were included in the “delta-group,” and children hospitalized between December 16th 2021 and February 28th 2022 were in the “omicron-group.”

Continuous data were expressed as median [interquartile range (IQR)], while categorical data were expressed as numbers and proportions (%). Descriptive statistics are presented for all study variables. We used Fisher's exact test or Pearson's chi-squared test (with Yates' continuity correction when necessary) to compare categorical and qualitative data and implemented the Wilcoxon rank sum test to evaluate continuous variables. A *p*-value of <5% was interpreted as evidence of a statistically significant difference. The analyses were performed using SAS software (version 9.4; Cary, NC, USA).

## Results

### Distribution of the Hospitalizations According to SARS-CoV-2 Waves and to Age

**Figure 1:**
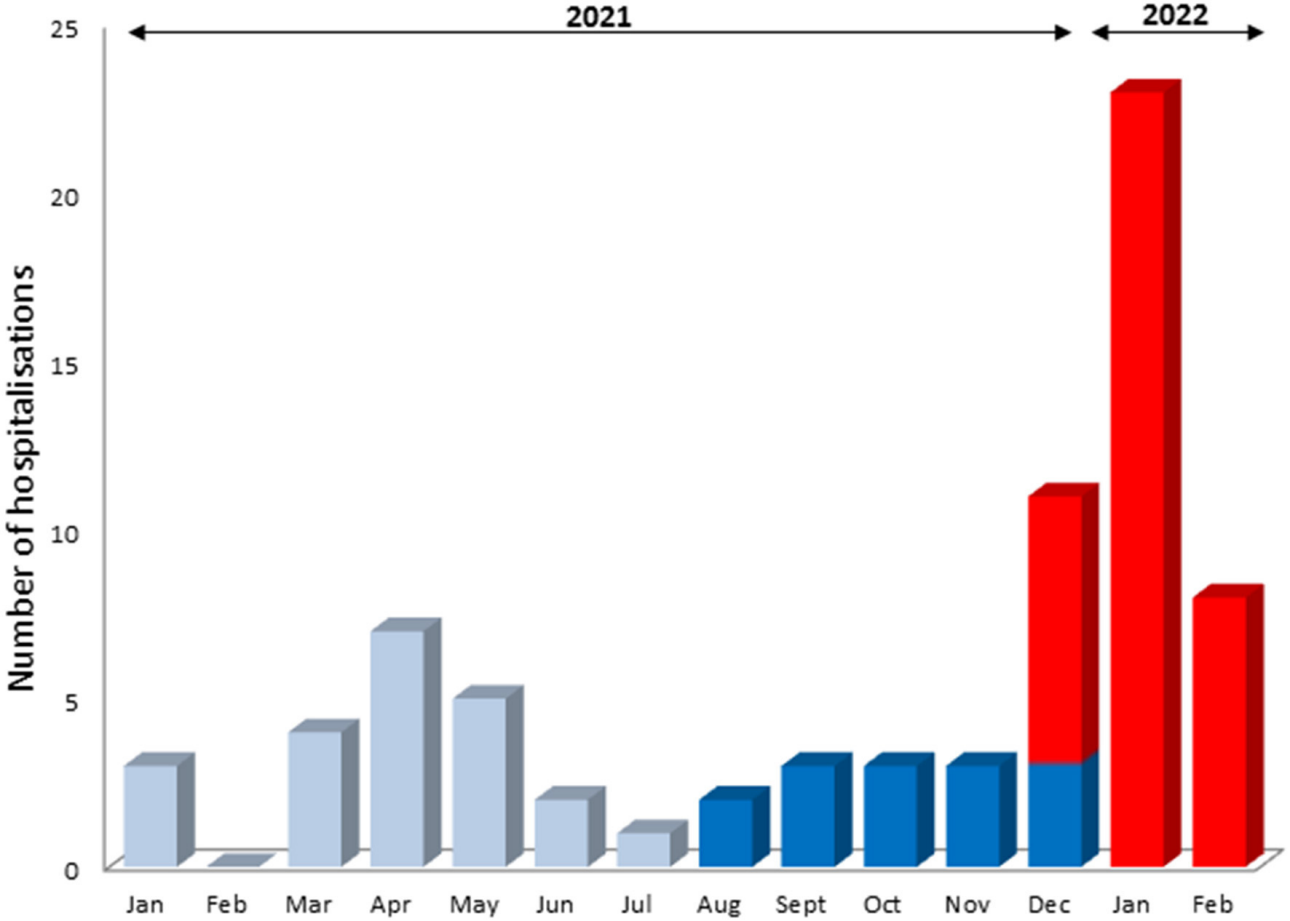
COVID-19 associated hospitalizations in children (January 2021–February 2022). Light blue (January 1st–August 31st 2021): alpha, beta, and gamma waves; deep blue (August 1st–December 15th 2021): delta wave; red (December 15th 2021–February 28th 2022): omicron wave.

The total number of children hospitalized monthly for COVID-19 between 1 January 2021 and 28 February 2022 is presented in Figure 1. During the study period (July 15th 2021 to February 28th 2022), 53 children aged 0–18 years were hospitalized for COVID-19 in our Pediatric Pulmonology Department. Among them, 14 (26.4%) were included in the delta group and 39 (73.6%) in the omicron group, while the duration of the study period was double that of the omicron wave. Indeed, the first group extended over 5 months (i.e., from July 15th to December 15th, 2021), whereas the second only over 2.5 months (i.e., from December 15th 2021 to February 28th 2022). While all of the 53 included children had positive PCR for SARS-CoV-2 in nasopharyngeal swab, only part of the SARS-CoV-2 variants were identified by Novaplex™ SARS-CoV-2 Variants I and IV Assays (Seegene, South Korea). Among the 14 children of the delta group, 7 (50%) were confirmed SARS-CoV-2 delta variant; and among the 39 children of the omicron group, 22 (56%) were confirmed SARS-CoV-2 omicron variant.

**Figure 2:**
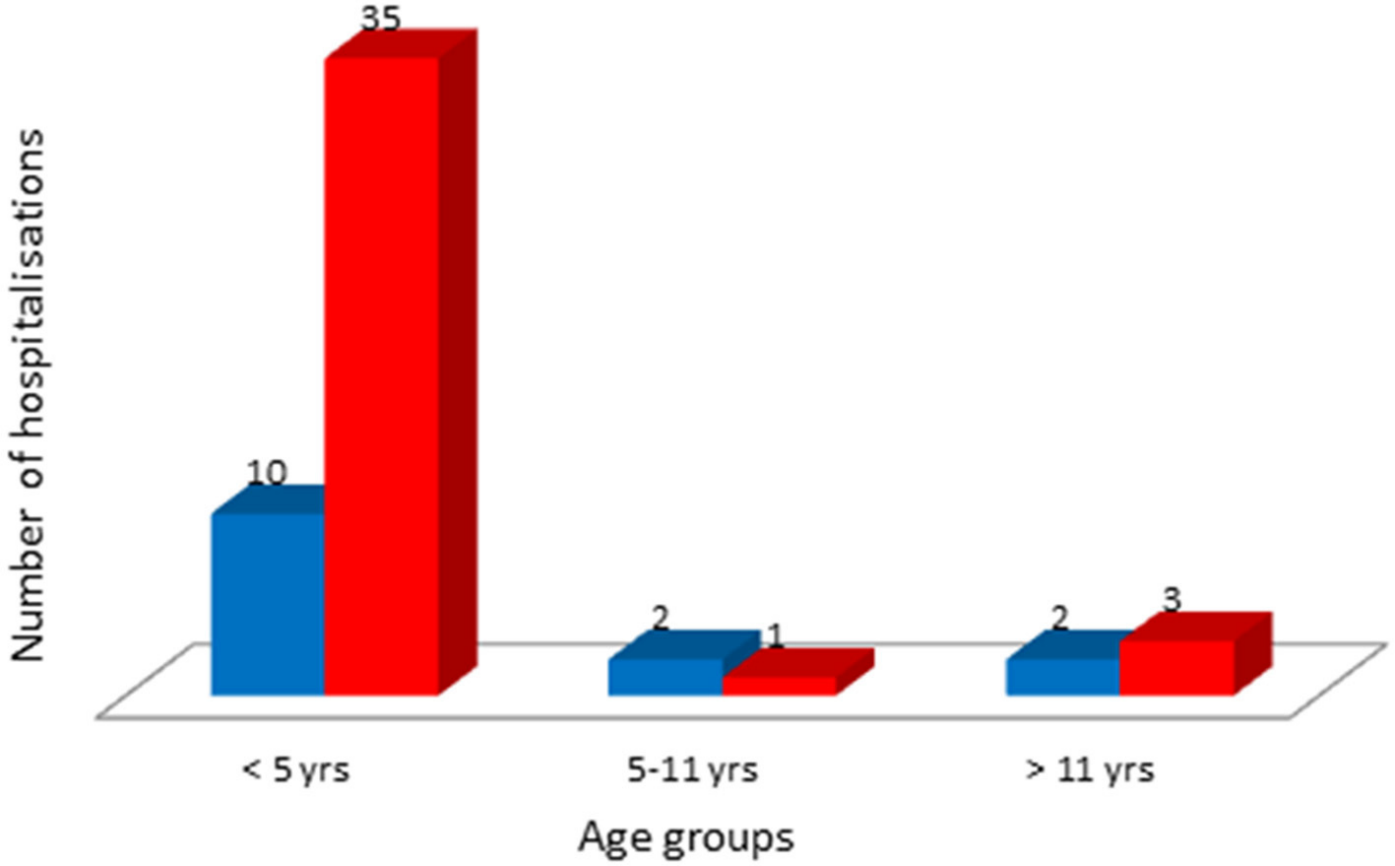
Distribution of children hospitalized for COVID-19 per age class. Deep blue (August 1st–December 15th 2021): delta wave; red (December 15th 2021–February 28th 2022): omicron wave.

The distribution of hospitalizations according to age group (<5, 5–11, and >11 years) is reported in Figure 2. Of the three patients in the omicron group who were older than 12 years, none were fully vaccinated. Two of them had not been vaccinated, and one had received an incomplete vaccination with only one injection 2 weeks prior to the onset of symptoms.

### Baseline Clinical Characteristics

The patients' baseline clinical characteristics according to the wave group are detailed in Table 1. During the omicron wave, hospitalized patients were mostly aged < 5 years (90 vs. 71% during the omicron and delta waves, respectively), and tended to have fewer pre-existing conditions (56 vs. 79% during the same period, respectively, *p* = 0.20). During the delta wave, 5/14 infants (36%) aged <3 months were hospitalized, compared to 8/39 (20%) during the omicron wave.

**Table 1:** General characteristics of the children hospitalized for COVID-19 during the delta and omicron waves.

	**Delta wave^a^**	**Omicron wave^b^**	***p*** **-value**
**Patients (** ***n*** **)**	14	39	
**Age (years): median [IQR]**	0.6 [0.1; 6.5]	0.6 [0.3; 1.6]	0.97
**Age class (** ***n*** **, %)**			0.18
<5 yrs	10 (71%)	35 (90%)	
> 5 yrs	4 (29%)	4 (10%)	
**Male (** ***n*** **, %)**	10 (71%)	26 (67%)	>0.99
**Pre-existing condition, i.e. underlying comorbidity or age < 3 months (** ***n*** **, %)**	11 (79%)	22 (56%)	0.14

a *Delta wave: from July 15th to December 15th, 2021*.

b *Omicron wave: from December 15th 2021 to February 28th 2022*.

In the delta group, the large majority of patients (11/14, 79%) had a pre-existing condition such as asthma, interstitial lung disease, congenital myopathy, obesity, Crohn's disease, and sickle cell disease; and 5 were infants under 3 months of age. In the omicron group, 22/39 (56%) children had an underlying condition: 7 had a respiratory disease (asthma, tuberculosis, cystic fibrosis, bronchodysplasia, interstitial lung disease, Langerhans histiocytosis, and Schwachman-Diamond syndrome), 2 a hematologic disease (sickle cell disease, Hodgkin's lymphoma), 2 a genetic disorder (Prader-Willi, CHARGE syndrome), one a cardiologic defect (pulmonary valvular stenosis), and 8 were infants under 3 months of age.

### Clinical Presentation and Explorations at COVID-19 Onset

The clinical presentation at COVID-19 onset is described in Table 2. The omicron variant caused significantly more digestive symptoms, such as diarrhea (33% vs. 0) during the omicron and delta waves respectively (*p* = 0.01), and refusal to eat (46 vs. 7%) during the omicron and delta waves respectively (*p* = 0.01). No patient in the omicron group presented with hemoptysis, compared to three patients (21%) in the delta group (*p* = 0.01). Body temperature was significantly higher during infections with the omicron variant than in those with the delta variant [39.2°C (38.9; 39.4) vs. 38.5°C (38.5; 38.7), respectively; *p* = 0.02]. Although not statistically significant, the proportion of children with poor symptom tolerance and deterioration of general health status was higher in the omicron group (59 vs. 36%) during the omicron and delta waves respectively, *p* = 0.21.

**Table 2:** Clinical presentation of the children hospitalized during the delta and omicron waves at COVID onset.

	**Delta wave^a^**	**Omicron wave^b^**	***p*** **-value**
**General symptoms**			
Fever (*n*, %)	11 (79%)	29 (74%)	>0.99
Poor fever tolerance (*n*, %)	2 (18%)	8 (28%)	>0.99
Fever duration [days, median (range)]	1 (1; 15)	2 (1; 9)	>0.99
Fatigue/deterioration of general status (n, %)	5 (36%)	23 (59%)	0.21
**Respiratory symptoms**			
Cough (*n*, %)	9 (64%)	26 (67%)	>0.99
Dyspnea (*n*, %)	9 (64%)	20 (51%)	0.53
Hemoptysis (*n*, %)	3 (21%)	0	0.02
**Upper respiratory airway**			
Acute rhinitis (*n*, %)	6 (43%)	23 (59%)	0.36
Pharyngitis (*n*, %)	1 (7%)	7 (18%)	0.67
Laryngitis (*n*, %)	0	3 (8%)	0.56
**Digestive symptoms**			
Vomiting (*n*, %)	1 (7%)	9 (23%)	0.26
Diarrhea (*n*, %)	0	13 (33%)	0.01
Refusal to eat (*n*, %)	1 (7%)	18 (46%)	0.01
**Initial vital signs**			
Highest temperature (°C) [median (IQR)]	38.5 [38.5; 38.7]	39.2 [38.9; 39.4]	0.03
Respiratory rate (/min) [median (IQR)]	42 [24; 54]	46 [35; 50]	0.53
Oxygen saturation (%)[median (IQR)]	98 [94; 99]	98 [95; 99]	0.93
Cardiac rate (/min) [median (IQR)]	135 [123; 151]	143 [133; 157]	0.31

a *Delta wave: from July 15th to December 15th, 2021*.

b *Omicron wave: from December 15th 2021 to February 28th 2022*.

Detailed information on the main explorations performed at admission is provided in Table 3. Six children (50%) were co-infected with other respiratory viruses during the delta wave and 22 (63%) during the omicron wave (details on the different viruses are provided in Table 3). Chest X-ray and thoracic CT-scan, when abnormal, were similar in both groups, with features of lung consolidation without specific localization. One patient in the delta group presented with pleural effusion and one in the omicron group with bilateral pneumothorax. No pulmonary embolisms were observed during these waves in our department.

**Table 3:** Results of the main explorations performed at admission of the children hospitalized for COVID-19 during the delta and omicron waves.

	**Delta wave^a^**	**Omicron wave^b^**	***p*** **-value**
**Nasopharyngeal result on PCR assay**			
Positive for SARS-CoV-2 (*n*, %)	14 (100%)	39 (100%)	
Positive for ≥1 other respiratory viruses (%)^*^	6/12 (50%)	22/35 (63%)	0.75
Respiratory Syncytial virus (*n*)	3	2	
Rhinovirus (*n*)	0	10	
Influenza virus (*n*)	0	3	
Parainfluenza (*n*)	2	2	
Coronavirus (*n*)	1	3	
Bocavirus (*n*)	0	6	
Adenovirus (*n*)	0	1	
Metapneumovirus (*n*)	0	1	
**Thoracic imaging**			
Chest radiography			
Number of patients, % of abnormal results	5 (60%)	26 (38%)	0.63
Computed tomography			
Number of patients, % of abnormal results	6 (100%)	3 (33%)	0.08
**Blood tests [median (IQR)]**			
WBC (x10^9^/L)	7.5 [4.5; 9.4]	10.5 [7.1; 14.3]	0.26
Lymphocytes (x10^9^/L)	2.4 [1.9; 2.6]	3.2 (1.4; 5.4]	0.32
Neutrophils (x10^9^/L)	1.5 [0.7; 5.4]	3.1 [1.8; 8.0]	0.26
Hemoglobin (g/dL)	11.5 [10.7; 12.5]	11.1 [10.0; 11.8]	0.29
Platelets (x10^9^/L)	318 [241; 387]	312 [256; 388]	0.73
CRP (mg/L)	8 [5; 20]	7 [0; 42]	0.72

* *These analyses were performed by means of Allplex Respiratory Panel Assays (Seegene)*.

a *Delta wave: from July 15th to December 15th, 2021*.

b *Omicron wave: from December 15th 2021 to February 28th 2022*.

### Management and Evolution

The management and clinical evolution are detailed in Table 4. Two patients (14%) required nutritional support during the delta wave and 11 (28%) during the omicron wave. During the latter period, the median [IQR] duration of nutritional support was 2 [1.25; 3] days. There were no differences in patient management or disease evolution between the two groups. Two patients (one during the delta wave and one during the omicron wave) were already on home oxygen therapy and non-invasive ventilation due to chronic respiratory insufficiency prior to hospitalization for COVID-19. For both, oxygen or ventilation needs increased respectively during 2 and 11 days before returning to previous support levels.

**Table 4:** Management and evolution of the children hospitalized for COVID during the delta and omicron waves.

	**Delta wave^a^**	**Omicron wave^b^**	***p*** **-value**
**Symptoms prior to hospitalization**						
Duration (days): median (IQR)	3 (2; 3.8)	3 (2, 4)	0.63
**Hospitalization duration (days): median (IQR)**	2.5 (2.0; 3.8)	3.0 (2.0; 4.8)	0.29
**Intensive Care Unit (** ***n*** **, %)**	0	4 (10%)	0.56
**Oxygen therapy (** ***n*** **, %)**	5 (36%)	12 (31%)	0.75
Oxygen therapy duration [days, median (IQR)]	2.0 (1.0; 2.0)	3 (1.5; 9.0)	0.18
**Non-invasive ventilation (** ***n*** **, %)**	0	3 (8%)	>0.99
**Invasive ventilation (** ***n*** **, %)**	0	2 (5%)	>0.99
**Nutritional support (** ***n*** **, %)**	2 (14%)	12 (31%)	0.31

a *Delta wave: from July 15th to December 15th, 2021*.

b *Omicron wave: from December 15th 2021 to February 28th 2022*.

## Discussion

This study compared the incidence and clinical symptoms of children hospitalized for COVID-19 during the delta and omicron waves. During the omicron wave, there was a major increase in the number of hospitalizations, with almost three times as many hospitalizations in half the time when compared to the delta variant, with the vast majority of children younger than 5 years. There were also distinct clinical characteristics, with higher temperature and poorly tolerated fever and a predilection for upper respiratory airways and digestive symptoms during the omicron wave.

According to the World Health Organization (WHO) data, the omicron variant has been responsible for five to six times more confirmed SARS-CoV-2 infections in Europe and America (1). Although it has been suggested that the omicron variant is associated with lower hospitalization rates due to a suspected reduction in disease severity, this wave has caused an important increase in the number of hospitalizations in children (1, 7, 11–13). This is in line with the surge in the number of hospitalizations for COVID-19 observed in our pediatric pulmonology department, which almost tripled between the two waves in half of the time. The higher infectivity of the omicron variant has been attributed to an exceptional number of mutations in the spike glycoprotein-binding human ACE2, resulting in increased infectivity of nasal epithelial cells and ACE2-positive cells (3, 17). These alterations in virus conformation influence antibody neutralization and facilitate viral immune escape, making it a variant of concern (3, 18).

In the light of these findings, questions have arisen regarding the vaccine efficacy. Although studies have suggested a decrease in vaccine-induced immunity after the second dose, others have shown that boosters can restore neutralizing immunity (19–21). Lauring et al. showed in adults that three doses of mRNA vaccine were necessary to obtain the same protection for the omicron variant as that provided for other variants after two doses (22). Similar results were observed in immunocompetent adolescents (12–17 years old), where vaccine efficacy toward the omicron variant was restored after three doses (23). In our study, none of the three patients older than 12 years hospitalized during the omicron wave were fully vaccinated. This result, along with the observation by others that children were more susceptible to infections/reinfections during the omicron wave despite vaccination or previous infection, requires that children be vaccinated when possible, and this includes the need for the booster dose (13, 24). This is supported by the 6-fold increase in the monthly hospitalization rate in non-vaccinated adolescents compared to that in vaccinated children during the omicron wave (13).

Along with the increased number of child hospitalizations, the symptoms observed at COVID-19 onset were somewhat different when subsequently infected by the omicron variant or by the delta and previous variants (12, 25–28). In Italian children, an analysis of online search trends suggested increased upper respiratory airway symptoms and possibly poorly tolerated fever, whereas dyspnea and anosmia/ageusia seemed less frequent (25). The latter finding could also be an indicator of the younger age of infected children and their inability to report such symptoms. Indeed, we found that hospitalized children were mostly aged under 5 years (90% during the omicron wave and 71% during the delta wave). We observed similar symptoms at the onset of infection to those reported by Cloete et al. in South African children (12). As such, we found that omicron caused significantly higher temperatures, diarrhea, and refusal to eat. The rate of underlying conditions was also in agreement with that reported by Cloete et al., with only 56% of the children hospitalized during the omicron wave vs. 79% during the delta wave. Similar to other studies, this study observed slightly more frequent upper respiratory airway symptoms during omicron waves (29, 30). The higher susceptibility to target upper airways could be a real concern in young children, a population prone to severe upper airway infections due to a smaller respiratory tract (29). A recent retrospective cohort study showed results similar to ours, with adults infected by the omicron variant being younger, with less frequent comorbidities and dyspnea, and more frequent upper respiratory airway symptoms (31). Finally, although these symptoms were not observed in this study, others have highlighted the risk of convulsions and venous cerebral thrombosis in children infected with the omicron variant (12, 15). Nevertheless, neurological signs have already been described in previous waves and should remain a cause of concern in children (27, 28).

The main limitation of this study is its retrospective and monocentric nature, which led to small number of inclusions. However, the scarce literature on the infections caused by the omicron variant in children makes it important to report the clinical features in the pediatric population and the specificities compared to previously described waves.

In conclusion, during the omicron wave, there was a major increase in the number of hospitalizations of children for COVID-19. These children were mostly under 5 years of age, younger than during previous waves (27). Unfortunately, children under 5 years of age cannot benefit from the vaccination as available SARS-CoV-2 vaccines are recommended for older children. Although the BNT162b2 COVID-19 vaccine has been deemed safe, immunogenic, and effective in preventing COVID-19 infection (16), concerns have arisen regarding the risk of myocarditis, especially in adolescents (32–35). This could explain why vaccination rates remain low in children aged 5–11 years old (36). For example in France, although the BNT162b2 COVID-19 vaccine is available for the children aged 5–11 years old since December 22, 2021, only 5% received at least one dose of as of April 29, 2022. Thus, describing the continuous evolution of COVID-19 symptoms and severity in children is essential for improving vaccination adherence.

## Data Availability Statement

The raw data supporting the conclusions of this article will be made available by the authors, without undue reservation.

## Ethics Statement

The studies involving human participants were reviewed and approved by the Local Ethics Committee of our institution, which waived the need for patients' consent (Study PED_COVID N°20200717191204). Written informed consent from the participants' legal guardian/next of kin was not required to participate in this study in accordance with the national legislation and the institutional requirements.

## Author Contributions

JT, BP, and HC were involved in the methodology, formal analysis, investigation, data curation, writing the original draft, reviewing and editing the manuscript, designing of tables and graphs, and verified the underlying data. AS, GA, LBe, LBi, AD-A, GT, and NN data were involved in data provision and reviewing and editing the manuscript. All authors had full access to all data in the study and accept responsibility for the decision to submit for publication.^1^

## Conflict of Interest

The authors declare that the research was conducted in the absence of any commercial or financial relationships that could be construed as a potential conflict of interest.

## Publisher's Note

All claims expressed in this article are solely those of the authors and do not necessarily represent those of their affiliated organizations, or those of the publisher, the editors and the reviewers. Any product that may be evaluated in this article, or claim that may be made by its manufacturer, is not guaranteed or endorsed by the publisher.

## Footnotes

^1^Le tableau de bord de la vaccination. Available online at: https://solidarites-sante.gouv.fr/grands-dossiers/vaccin-covid-19/article/le-tableau-de-bord-de-la-vaccination.

## References

[1] Belay ED Godfred-Cato S. SARS-CoV-2 spread and hospitalisations in paediatric patients during the omicron surge. Lancet Child Adolesc Health. (2022) 6:280–1. 10.1016/S2352-4642(22)00060-8 35189084 PMC8856665

[2] Islam MR Hossain MJ. Detection of SARS-CoV-2 Omicron (B11529) variant has created panic among the people across the world: what should we do right now? J Med Virol. (2022) 94:1768–9. 10.1002/jmv.27546 34939691

[3] Viana R Moyo S Amoako DG Tegally H Scheepers C Althaus CL. Rapid epidemic expansion of the SARS-CoV-2 Omicron variant in southern Africa. Nature. (2022) 603:679–86. 10.1038/s41586-022-04411-y 35042229 PMC8942855

[4] Santé Publique France. Chiffres clés et évolution de la COVID-19 en France et dans le Monde. Avalaible online at: https://www.santepubliquefrance.fr/dossiers/coronavirus-covid-19/ (accessed April 29, 2022).

[5] Taytard J Prevost B Corvol H. More on BNT162b2 Covid-19 vaccine in children 5 to 11 years of age. N Engl J Med. (2022) 386:2201556. 10.1056/NEJMc2201556 35235722

[6] Mahase E. Covid-19: hospital admission 50-70% less likely with omicron than delta, but transmission a major concern. BMJ. (2021) 375:n3151. 10.1136/bmj.n3151 34952835

[7] Wolter N Jassat W Walaza S Welch R Moultrie H Groome M. Early assessment of the clinical severity of the SARS-CoV-2 omicron variant in South Africa: a data linkage study. Lancet. (2022) 399:437–46. 10.1016/S0140-6736(22)00017-4 35065011 PMC8769664

[8] Collie S Champion J Moultrie H Bekker LG Gray G. Effectiveness of BNT162b2 vaccine against omicron variant in South Africa. N Engl J Med. (2022) 386:494–6. 10.1056/NEJMc2119270 34965358 PMC8757569

[9] Maslo C Friedland R Toubkin M Laubscher A Akaloo T Kama B. Characteristics and outcomes of hospitalized patients in South Africa during the COVID-19 omicron wave compared with previous waves. J Am Med Assoc. (2022) 327:583–4. 10.1001/jama.2021.24868 34967859 PMC8719272

[10] Lima DGS Figueiredo TMR Pereira YTG Almino M Pereira LM de Menezes HL. The effects of the silence on south African children and adolescents against a global alert on the newly identified coronavirus variant: Omicron. J Pediatr Nurs. (2021) 2021:S0882–596300365-1. 10.1016/j.pedn.2021.11.032 34930655 PMC9257976

[11] Shi DS Whitaker M Marks KJ Anglin O Milucky J Patel K. Hospitalizations of children aged 5-11 years with laboratory-confirmed COVID-19 - COVID-NET, 14 States, March 2020-February 2022. Morb Mortal Wkly Rep. (2022) 71:574–81. 10.15585/mmwr.mm7116e1 35446827 PMC9042359

[12] Cloete J Kruger A Masha M du Plessis NM Mawela D Tshukudu M. Paediatric hospitalisations due to COVID-19 during the first SARS-CoV-2 omicron (B11529) variant wave in South Africa: a multicentre observational study. Lancet Child Adolesc Health. (2022) 6:294–302. 10.1016/S2352-4642(22)00027-X 35189083 PMC8856663

[13] Marks KJ Whitaker M Anglin O Milucky J Patel K Pham H. Hospitalizations of children and adolescents with laboratory-confirmed COVID-19 - COVID-NET, 14 States, July 2021-January 2022. Morb Mortal Wkly Rep. (2022) 71:271–8. 10.15585/mmwr.mm7107e4 35176003 PMC8853476

[14] Ludvigsson JF. Convulsions in children with COVID-19 during the Omicron wave. Acta Paediatr. (2022) 111:1023–6. 10.1111/apa.16276 35098577 PMC9303202

[15] Vallejo S Mendez-Echevarria A Del Rosal T Falces-Romero I Muñoz-Caro JM Buitrago Sánchez NM. Omicron and thrombosis in children: cause for concern? Pediatr Infect Dis J. (2022) 41:e252–e4. 10.1097/INF.0000000000003501 35213862 PMC8997015

[16] Walter EB Talaat KR Sabharwal C Gurtman A Lockhart S Paulsen GC. Evaluation of the BNT162b2 Covid-19 vaccine in children 5 to 11 years of age. N Engl J Med. (2022) 386:35–46. 10.1056/NEJMoa2116298 34752019 PMC8609605

[17] Zhang Y Zhang T Fang Y Liu J Ye Q Ding L. SARS-CoV-2 spike L452R mutation increases Omicron variant fusogenicity and infectivity as well as host glycolysis. Signal Transduct Target Ther. (2022) 7:76. 10.1038/s41392-022-00941-z 35264568 PMC8905570

[18] Cui Z Liu P Wang N Wang L Fan K Zhu Q. Structural and functional characterizations of infectivity and immune evasion of SARS-CoV-2 Omicron. Cell. (2022) 185:860–71.e13. 10.1016/j.cell.2022.01.019 35120603 PMC8786603

[19] Dejnirattisai W Huo J Zhou D Zahradník J Supasa P Liu C. SARS-CoV-2 Omicron-B.1.1.529 leads to widespread escape from neutralizing antibody responses. Cell. (2022) 185:467–84.e15. 10.1016/j.cell.2021.12.046 35081335 PMC8723827

[20] Edara VV Manning KE Ellis M Lai L Moore KM Foster SL. mRNA-1273 and BNT162b2 mRNA vaccines have reduced neutralizing activity against the SARS-CoV-2 omicron variant. Cell Rep Med. (2022) 3:100529. 10.1016/j.xcrm.2022.100529 35233550 PMC8784612

[21] Garcia-Beltran WF St Denis KJ Hoelzemer A Lam EC Nitido AD Sheehan ML. mRNA-based COVID-19 vaccine boosters induce neutralizing immunity against SARS-CoV-2 Omicron variant. Cell. (2022) 185:457–66.e4. 10.1016/j.cell.2021.12.033 34995482 PMC8733787

[22] Lauring AS Tenforde MW Chappell JD Gaglani M Ginde AA McNeal T. Clinical severity of, and effectiveness of mRNA vaccines against, covid-19 from omicron, delta, and alpha SARS-CoV-2 variants in the United States: prospective observational study. BMJ. (2022) 376:e069761. 10.1136/bmj-2021-069761 35264324 PMC8905308

[23] Klein NP Stockwell MS Demarco M Gaglani M Kharbanda AB Irving SA. Effectiveness of COVID-19 Pfizer-BioNTech BNT162b2 mRNA vaccination in preventing COVID-19-associated emergency department and urgent care encounters and hospitalizations among nonimmunocompromised children and adolescents aged 5-17 years - VISION Network, 10 States, April 2021-January 2022. Morb Mortal Wkly Rep. (2022) 71:352–8. 10.15585/mmwr.mm7109e3 35239634 PMC8893336

[24] Chen LL Chua GT Lu L Chan BP Wong JS Chow CC. Omicron variant susceptibility to neutralizing antibodies induced in children by natural SARS-CoV-2 infection or COVID-19 vaccine. Emerg Microbes Infect. (2022) 11:543–7. 10.1080/22221751.2022.2035195 35084295 PMC8843159

[25] Lippi G Nocini R Henry BM. Analysis of online search trends suggests that SARS-CoV-2 Omicron (B.1.1.529) variant causes different symptoms. J Infect. (2022) 2022:1214484. 10.21203/rs.3.rs-1214484/v1 35183609 PMC8851877

[26] Nathan N Prevost B Lambert S Schnuriger A Corvol H. Severe acute respiratory syndrome coronavirus 2 variant delta infects all 6 siblings but spares comirnaty (BNT162b2, BioNTech/Pfizer)-vaccinated parents. J Infect Dis. (2021) 224:1984–6. 10.1093/infdis/jiab410 34409999 PMC8499735

[27] Nathan N Prevost B Sileo C Richard N Berdah L Thouvenin G. The wide spectrum of COVID-19 clinical presentation in children. J Clin Med. (2020) 9:92950. 10.3390/jcm9092950 32932612 PMC7564665

[28] Nathan N Prevost B Corvol H. Atypical presentation of COVID-19 in young infants. Lancet. (2020) 395:1481. 10.1016/S0140-6736(20)30980-6 32353326 PMC7185921

[29] Brewster RCL Parsons C Laird-Gion J Hilker S Irwin M Sommerschield A. COVID-19-associated croup in children. Pediatrics. (2022) 2022:56492. 10.1542/peds.2022-056492 35257175

[30] Murata Y Tomari K Matsuoka T. Children with croup and SARS-CoV-2 infection during the large outbreak of Omicron. Pediatr Infect Dis J. (2022) 41:e249. 10.1097/INF.0000000000003484 35185142 PMC8997017

[31] Bouzid D Visseaux B Kassasseya C Daoud A Fémy F Hermand C. Comparison of patients infected with delta versus Omicron COVID-19 variants presenting to paris emergency departments : a retrospective cohort study. Ann Intern Med. (2022) 2022 :308. 10.7326/M22-0308 35286147 PMC8941485

[32] Hause AM Gee J Baggs J Abara WE Marquez P Thompson D. COVID-19 vaccine safety in adolescents aged 12-17 years - United States, December 14, 2020-July 16, 2021. Morb Mortal Wkly Rep. (2021) 70:1053–8. 10.15585/mmwr.mm7031e1 34351881 PMC8367318

[33] Lai FTT Chua GT Chan EWW Huang L Kwan MYW Ma T. Adverse events of special interest following the use of BNT162b2 in adolescents: a population-based retrospective cohort study. Emerg Microbes Infect. (2022) 11:885–93. 10.1080/22221751.2022.2050952 35254219 PMC8942549

[34] Oster ME Shay DK Su JR Gee J Creech CB Broder KR. Myocarditis cases reported after mRNA-based COVID-19 vaccination in the US From December 2020 to August 2021. J Am Med Assoc. (2022) 327:331–40. 10.1001/jama.2021.24110 35076665 PMC8790664

[35] Mevorach D Anis E Cedar N Hasin T Bromberg M Goldberg L. Myocarditis after BNT162b2 vaccination in Israeli adolescents. N Engl J Med. (2022) 386:998–9. 10.1056/NEJMc2116999 35081295 PMC8823652

[36] Kim C Yee R Bhatkoti R Carranza D Henderson D Kuwabara SA. COVID-19 vaccine provider access and vaccination coverage among children aged 5-11 years - United States, November 2021-January 2022. Morb Mortal Wkly Rep. (2022) 71:378–83. 10.15585/mmwr.mm7110a4 35271559 PMC8911999

